# Gestational cytokine concentrations and neurocognitive development at 7 years

**DOI:** 10.1038/s41398-018-0112-z

**Published:** 2018-03-13

**Authors:** Akhgar Ghassabian, Paul S Albert, Mady Hornig, Edwina Yeung, Sara Cherkerzian, Risë B Goldstein, Stephen L Buka, Jill M Goldstein, Stephen E Gilman

**Affiliations:** 1 Departments of Pediatrics, Environmental Medicine, and Population Health, New York University School of Medicine, New York, NY USA; 2 Epidemiology Branch, Division of Intramural Population Health Research, Eunice Kennedy Shriver National Institute of Child Health and Human Development, National Institutes of Health, Bethesda, MD USA; 3 Biostatistics and Bioinformatics Branch, Division of Intramural Population Health Research, Eunice Kennedy Shriver National Institute of Child Health and Human Development, National Institutes of Health, Bethesda, MD USA; 4 Biostatistics Branch, Division of Cancer Epidemiology and Genetics, National Cancer Institute, National Institutes of Health, Bethesda, MD USA; 5 Center for Infection and Immunity, Columbia University Mailman School of Public Health, New York, NY USA; 6 Department of Epidemiology, Columbia University Mailman School of Public Health, New York, NY USA; 7 Department of Pediatric Newborn Medicine, Brigham and Women’s Hospital, Boston, MA USA; 8 Departments of Psychiatry and Medicine, Harvard Medical School, Boston, MA USA; 9 Health Behavior Branch, Division of Intramural Population Health Research, Eunice Kennedy Shriver National Institute of Child Health and Human Development, National Institutes of Health, Bethesda, MD USA; 10 Department of Epidemiology, Brown University School of Public Health, Providence, RI USA; 11 Division of Psychiatric Neuroscience, Department of Psychiatry, Massachusetts General Hospital, Boston, MA USA; 12 Department of Mental Health, The Johns Hopkins Bloomberg School of Public Health, Baltimore, MD USA

## Abstract

Gestational inflammation may contribute to brain abnormalities associated with childhood neuropsychiatric disorders. Limited knowledge exists regarding the associations of maternal cytokine levels during pregnancy with offspring neurocognitive development. We assayed the concentrations of five cytokines (interleukin (IL)-6, IL-1β, IL-8, tumor necrosis factor alpha (TNF-α), and IL-10) up to four times in the 2nd and 3rd trimesters of pregnancy using stored prenatal sera from 1366 participants in the New England Family Study (enrollment 1959–1966). Intelligence (IQ), academic achievement, and neuropsychological functioning of singleton offspring were assessed at age 7 years using standardized tests. We used linear mixed models with random effects to estimate the cumulative exposure to each cytokine during 2nd and 3rd trimesters, and then related cumulative cytokine exposure to a wide range of offspring neurocognitive outcomes. We found that children of women with higher levels of the pro-inflammatory cytokine, TNF-α, in the 2nd and 3rd trimesters had lower IQ (*B* = −2.51, 99% CI: −4.84,−0.18), higher problem scores in visual-motor maturity (*B* = 0.12, 99% CI: 0.001,0.24), and lower Draw-a-Person test scores (*B* = −1.28, 99% CI: −2.49,−0.07). Higher gestational levels of IL-8, another pro-inflammatory molecule, were associated with better Draw-a-Person test scores and tactile finger recognition scores. Other cytokines were not associated with our outcome of interest. The opposing directions of associations observed between TNF-α and IL-8 with childhood outcomes suggest pleiotropic effects of gestational inflammation across the domains of neurocognitive functioning. Although the path to psychopathological disturbances in children is no doubt multifactorial, our findings point to a potential role for immune processes in the neurocognitive development of children.

## Introduction

Maternal immune system activation during pregnancy—whether in response to an infection, autoimmune processes, stress, or a genetic predisposition—may contribute to the brain abnormalities associated with neuropsychiatric disorders such as autism spectrum disorder (ASD)^1,2^. Experimental studies in animals suggest that immune activation across gestation is associated with aberrant neurogenesis, neural migration, and synaptogenesis in the offspring^3,4^. Interruptions in these processes in animal models contribute to neuropathological abnormalities including reduced cortical thickness, smaller volumes in specific brain regions (e.g., hippocampus), and abnormalities in brain connectivity and plasticity. Neurochemical alterations are also reported as a consequence of immune activation during gestation^3,4^.

Epidemiological studies have shown that even low-grade inflammation in pregnancy might impact the developing fetal brain, but results are inconsistent. For example, the presence of fever during pregnancy—a sign of systemic inflammation—was associated with increased risk of ASD among offspring (particularly with 2nd trimester fever)^5^. Brown et al. found that higher levels of maternal C-reactive protein (CRP) in the 1st and 2nd trimesters of pregnancy were associated with higher risk of ASD^6^. In contrast, however, another study found that maternal mid-pregnancy levels of CRP were lower in mothers of children with ASD as compared with controls^7^. In these studies, maternal inflammatory molecules were assessed at one single time point. To date, few studies have examined inflammatory markers in pregnant women on multiple occasions during gestation^8,9^. Moreover, little is known regarding the role of immune molecules in promoting healthy neurocognitive development throughout gestation. Across pregnancy, hormonal changes in the mother along with the production of protective factors by the growing fetus that are required to achieve successful embryo implantation and fetal development lead to critical variations in T-cell balance and inflammatory markers, even during the course of normal gestation^10^. Therefore, a single measurement of gestational inflammatory markers may not adequately reflect cumulative fetal exposure to potentially neurotoxic (or neuroprotective) exposures during critical periods of brain development.

To the extent that gestational inflammation causes deviations from typical neurodevelopmental trajectories that result in elevated risk of neuropsychiatric disorders^11–13^, it is unlikely that normal neurocognitive functioning in childhood would remain otherwise intact. This is important because impairments such as intellectual abilities, language, and higher order cognitive processes are frequent manifestations of childhood psychiatric disorders and also exacerbate the course of adult psychiatric disorders^14^. Perturbation in gestational immune activity might therefore have broad consequences for offspring neurodevelopment ranging from neurological abnormalities and deficits in executive function^15^ in addition to raising the risk of the development of a range of neuropsychiatric diagnoses. However, evidence supporting this hypothesis is limited^16^.

Accordingly, we measured concentrations of five cytokines (interleukin (IL)-1β, IL-8, IL-6, IL-10, and tumor necrosis factor (TNF)-α) previously linked to psychopathologic disturbances in offspring^17^ during the 2nd and 3rd trimester of pregnancy in a population-based cohort of pregnant women. Receptors for these cytokines are present in several key brain regions including the hippocampus, the amygdala, and both ventromedial and paraventricular hypothalamic nuclei^18,19^. We selected IL-10 because of its anti-inflammatory properties. Using a two-stage analytic process, we first derived a measure of the cumulative cytokine concentration and then related these cumulative concentrations to offspring’s cognitive performance, academic achievement, and neuropsychological functioning at age 7 years. The study design builds upon prior work by including repeated measures of cytokines across pregnancy rather than relying solely on a single time measurement, and investigating a spectrum of neurocognitive outcomes in children that have been implicated in a broad range of neuropsychiatric diagnoses as adults.

## Materials and methods

Participants were offspring of 1366 singleton pregnancies enrolled in the Collaborative Perinatal Project (CPP, 1959–1966)^13,16^, who were identified based on their participation in one or more adult follow-up studies of the Boston and Providence CPP, known as the New England Family Study (NEFS). Maternal serum samples were collected serially during pregnancy and stored in the repositories. We included mother–child pairs with one or more assays of gestational cytokines (beginning week 14 of gestation until delivery, *n* = 2366 assays). Among 1366 singleton pregnancies, 101 pregnant women contributed with two children, six with three children, and two with four children. Seventy-six percent of prenatal serum samples (*n* = 1847) were drawn between weeks 14 and 34 of gestation. In the 1366 pregnancies included, there were 549 women for whom IL-1β was measured only once (40.2%), 654 for whom it was measured twice (47.9%), 145 for whom it was measured three times (10.6%), and 18 for whom it was measured four times (Supplementary Table 1). Women with one, two, three, or four assays of IL-1β were comparable in their characteristics including age, weight and height, history of treatment for psychiatric disorders, preeclampsia, and gestational diabetes (data not shown); with the exception that women who had cytokine data from a single time point were more likely to have high socioeconomic disadvantage (21.8%) compared to women with two (14.3%), three (12.4%), or four assays (5.6%). Information on neurocognitive functioning was available for 1128 mother–child pairs (83% of 1366).

We assayed stored maternal serum for concentrations of IL-1β, IL-8, IL-6, IL-10, and TNF-α using multiplexed immunoassays. Cytokine levels were assessed using a multiplexed, bead-based immunoassay (Milliplex^TM^ human cytokine panel, MPXHCYTO-60K, Millipore, MO) on a Luminex 3D^TM^ detection platform (Luminex Corporation, TX)^20^. Assay sensitivities ranged from 0.1–0.4 pg/mL. Twenty-five microliters of each serum sample were diluted 1:1 in assay buffer and run on the same plate with six serial dilutions (3.2–10,000 pg/mL) of cytokine standards buffer only (background), two commercial quality control samples, and one in-house plasma sample on each 96-well plate. Samples were treated similarly (same number of freeze–thaw cycles), using assay kits and reagents from a single lot, and all assays were completed within a 2-month period. All samples were run in duplicate and mean values were reported. Samples yielding problematic data (bead count errors or elevated inter-well covariance (CV); ~5% of samples) were re-assayed using additional previously prepared, assay-size, frozen aliquots, minimizing the number of freeze–thaw cycles for any required repeats. Assays were completed according to manufacturers’ protocols, with overnight incubation at 4 °C on a shaker prior to detection of mean fluorescence intensities (MFI) of analyte-specific immunoassay beads on the Luminex 3D. Raw data (MFI) were captured using Luminex xPONENT™ software (v.4.0.846.0) and concentrations of immune factors in each sample were interpolated from standard curves using a 5-parameter, weighted, logistic regression curve equation in Milliplex Analyst™ (v.3.5.5.0). To minimize potential bias, all study samples were assigned randomly across all assay plates. Inter-assay CV was controlled by adherence to stringent protocols, maximizing reliability, and comparing data across plates from replicates of serial standard curves, kit-specific quality control samples, and in-house plasma control samples that were included on each plate. The laboratory’s intra-assay CV was within the manufacturer’s range of <10%. Measurements below the lower limit of detection of any analyte were recoded to the midpoint between zero and the limit of detection for that analyte. Samples initially yielding values at or above the upper limit of analyte detection were re-assayed at multiple serial dilutions to bring concentrations into detectable (linear) range. Several previous studies using samples stored under similar conditions and for a similar length of time (>40 years) demonstrated the long-term stability of cytokines and reported associations of cytokine levels with several outcomes in the offspring^11,13,16^.

Study psychologists evaluated children’s neurocognitive function at age 7 years. Assessments were performed following a strict protocol and extensive quality controls. Verbal and performance IQ were assessed using the Wechsler Intelligence Scale for Children (WISC)^21^ and the full-scale IQ was calculated based on a combination of these two scores. Children’s academic performance was assessed using the Wide Range Achievement Test (WRAT)^22^. Based on the WRAT, children received scores in three domains of reading, spelling, and arithmetic skills that represented their rating as compared to their grade placement. The Bender Gestalt test is a brief test of visual-motor skills appropriate for children of 3 years and older and was used to assess visual-motor functioning^23^. Higher Bender Gestalt test scores indicated more problems. Psychologists also administered the Goodenough-Harris Draw-a-Person Test (evaluating performance abilities; higher scores indicate better performance) and Tactile Finger Recognition (evaluating motor functioning, higher scores indicate better performance) to children^24,25^.

Information on maternal age at enrollment, educational level attained, and race was collected from women during study enrollment by in-person interview^16^. Participants also reported on the household income, parental occupation, and family structure (both parents at home, single, or divorced/separated /widowed). A socioeconomic disadvantage score was calculated on the basis of parental education and occupation, household income, and family structure (higher scores defined worse disadvantage)^26^. Women reported on the number of cigarettes smoked per day in pregnancy and a history of treatment for psychiatric disorders. Presence of gestational diabetes and preeclampsia was recorded on obstetric diagnostic summaries. Maternal body mass index (BMI) was calculated using self-reported pre-pregnancy weight and height.

We used a two-stage regression calibration approach for analysis that summarized the repeated concentration measures of each cytokine during pregnancy in the first stage; in the second stage, we related measures of cumulative cytokine exposure to childhood neurocognitive function. This method accounts for any measurement error in the cytokine assays^27^, and for variability in cytokine concentrations across gestation^9^. An additional advantage of this approach is that cumulative inflammatory burden can be estimated for each pregnancy even though there might be a sizable fraction of pregnancies with only a single measurement.

First, the cumulative concentration of each cytokine (log-transformed) across the 2nd and 3^rd^ trimesters was estimated using linear piecewise mixed models. Linear piecewise mixed models were fitted with a pregnancy-level random intercept to estimate the variation in the concentration of each cytokine across pregnancy^28^. The piecewise mean structure incorporated a linear term from early in the 2nd trimester through the end of that trimester, an intercept centered at week 27 marking the beginning of the 3rd trimester; a linear term from the beginning of the 3rd trimester through delivery; and an indicator variable (*D*) that indicated whether any sera were obtained on the day of delivery for that pregnancy (to account for cytokine variations associated with parturition^29^). For each pregnancy, parameters from these models were used to define two lines joined at mid-gestation, the area below these lines used to quantify a mother’s cumulative inflammatory burden over pregnancy.

Second, we examined the association between log-transformed estimates of the cumulative cytokine concentrations across 2nd and 3rd trimesters and the offspring’s neurocognitive outcomes. We used linear regression to analyze the continuous outcomes and regression coefficients with 99% confidence intervals (CI) (two-sided test with *α* = 0.01, a Bonferroni correction of *p* = 0.05/5 cytokines to account for multiple cytokine tested). For significant associations involving pro-inflammatory cytokines in our panel, analyses were also performed with the ratio of pro-inflammatory cytokines to that of the anti-inflammatory cytokine IL-10. Neurocognitive scores with a skewed distribution were log-transformed (WRAT arithmetic score, Bender Gestalt score, and Tactile Finger Recognition score). Models were adjusted for the child’s sex and age at neurocognitive assessment, and maternal race, education, age at study enrollment, socioeconomic disadvantage, cigarettes smoked during pregnancy, history of treatment for psychiatric disorders, BMI prior to pregnancy, preeclampsia, and gestational diabetes, as these factors are commonly associated with gestational inflammation and early neurodevelopment^6,8,11,30^. Supplementary Tables 2 and 3 present the associations of covariates with cumulative cytokine concentrations. Analyses of offspring outcomes were conducted using generalized estimating equations (GEE) to account for the presence of 109 sibling sets in the analysis sample. To examine whether effects might be restricted to the extremes of the distributions, we ran the same models with cumulative cytokine measures categorized into quintiles. As some studies suggested a sex-specific effect of cytokines in relation to early neuropsychiatric outcomes^11,13^, an interaction with sex was tested in relation to neurodevelopmental outcomes. Analyses were conducted in SAS 9.4.

## Results

Characteristics of the 1366 pregnant women and their children as well as means and standard deviations of the cumulative measures of cytokines are presented in Table 1. The mean age of study enrollment was 24.8 years (SD = 5.7), the sample was predominantly White (88.8%), and a very few pregnancies had preeclampsia or gestational diabetes.

**Table 1:** Participants’ characteristics (*n* = 1366)

Maternal characteristics	Mean	SD
Age at enrollment, year	24.8	5.7
Race		
White	1213	88.8
Non-White	153	11.2
Prenatal socioeconomic disadvantage		
0–1	677	49.5
1.5–2.5	457	33.5
≥3	232	17.0
History of treatment for psychiatric disorders, yes	155	11.3
Preeclampsia, yes	20	1.5
Gestational diabetes, yes	13	0.9
Education, year	11.5	3.2
Pre-pregnancy body mass index	22.9	4.2
Maximum number of cigarette per day in pregnancy	7	0, 20
Cumulative cytokine concentration in pregnancy		
IL-1β	64.0	167.5
IL-8	804.4	1405.8
IL-6	45.2	108.0
IL-10	67.8	316.7
TNF-α	91.5	41.5
Child characteristics		
Verbal IQ score	100.0	13.9
Performance IQ score	104.4	13.9
Total IQ score	102.0	13.9
Bender Gestalt total raw score	6	4, 8
Goodenough-Harris Drawing raw score	21.8	6.3
Tactile Finger Recognition total score	9	9–10
WRAT spelling raw score	27.4	5.9
WRAT reading raw score	41.5	11.7
WRAT arithmetic raw score	22	20, 24

Numbers are mean (SD) for continuous normally distributed variables, median (25th, 75th quartiles) for continuous variables with skewed distribution (Bender Gestalt total raw score, Tactile Finger Recognition total score, and WRAT arithmetic raw score) and *n* (percentage) for categorical variables

*IL* interleukin, *IQ* intelligence quotient, *SD* standard deviation, *TNF* tumor necrosis factor, *WRAT* Wide Range Achievement Test

The first stage of the analyses involved fitting linear mixed models of the concentrations of each cytokine during pregnancy across 2nd and 3rd trimesters of pregnancy (coefficients from which are presented in Supplementary Table 4). These coefficients were then used to derive cumulative concentration measures for each pregnancy, which were then related to children’s neurocognitive outcomes at age 7 (Tables 2–4). There was a negative association between gestational TNF-α and children’s IQ scores: for each unit difference in the cumulative concentration of gestational TNF-α, full-scale IQ scores were, on average, 2.51 points lower (99% CI: −4.84, −0.18). This association was most prominent in the domain of performance IQ (Table 2). There were no associations between TNF-α, IL-10 ratio, and children’s IQ. We found no association between any measure of gestational inflammation and children’s academic achievement scores (Table 3). In the domain of neuropsychological functioning (Table 4), higher concentrations of gestational TNF-α were associated with higher problem scores on the test of concept formation (*B* = 0.12, 99% CI: 0.001, 0.24) and poorer performance on the Goodenough-Harris Drawing task (*B* = −1.28, 99% CI: −2.49, −0.07). In contrast, *higher* concentrations of gestational IL-8 were associated with better performance on the Drawing task (*B* = 0.51, 99% CI: 0.11, 0.90) and on the Tactile Finger Recognition Task (*B* = 0.02, 99%: 0.01, 0.04). No associations between IL-8, IL-10 ratio, and neuropsychological functioning were observed.

**Table 2:** Gestational cytokine concentration in the 2nd and 3rd trimesters and children’s IQ at 7 years (*n* = 1128)

	Full-scale IQ score		Verbal-scale IQ score		Performance-scale IQ score	
	*B*	99% CI	*B*	99% CI	*B*	99% CI
IL-1β	−0.38	−1.13, 0.38	−0.21	−1.05, 0.63	−0.45	−1.29, 0.38
IL-8	0.42	−0.33, 1.17	0.39	−0.45, 1.23	0.38	−0.42, 1.18
IL-6	−0.05	−1.18, 1.07	0.42	−0.74, 1.57	−0.50	−1.82, 0.73
IL-10	−0.28	−1.50, 0.95	0.52	−0.80, 1.84	−1.07	−2.40, 0.26
TNF-α	−2.51	−4.84, −0.18	−1.52	−3.90, 0.85	−3.00	−5.59, −0.38

Models are adjusted for child sex and age, and maternal race, education, age, socioeconomic disadvantage, smoking in pregnancy, history of treatment for psychiatric disorders, body mass index, preeclampsia, and gestational diabetes

Effect sizes are reported per log unit increase in the cumulative cytokine concentration (pg/mL) of each cytokine during gestation

*CI* confidence interval, *IL* interleukin, *TNF* tumor necrosis factor

**Table 3:** Gestational cytokine concentration in the 2nd and 3rd trimesters and children’s academic achievement at 7 years (*n* = 1128)

	WRAT spelling score		WRAT reading score		WRAT arithmetic score	
	*B*	99% CI	*B*	99% CI	*B*	99% CI
IL-1β	−0.02	−0.37, 0.32	0.03	−0.67, 0.73	0.00	−0.01, 0.01
IL-8	0.09	−0.26, 0.45	−0.28	−0.97, 0.41	−0.001	−0.01, 0.01
IL-6	−0.14	−0.61, 0.32	−0.45	−1.39, 0.49	0.002	−0.01, 0.02
IL-10	−0.04	−0.55, 0.47	−0.24	−1.29, 0.81	−0.01	−0.02, 0.01
TNF-α	−0.15	−1.22, 0.92	−1.53	−3.69, 0.63	−0.02	−0.05, 0.01

Models are adjusted for a child’s sex and age, and maternal race, education, age, socioeconomic disadvantage, history of treatment for psychiatric disorders, body mass index, preeclampsia, and gestational diabetes

Effect sizes are reported per log unit increase in the cumulative cytokine concentration (pg/mL) of each cytokine during gestation

WRAT arithmetic score was log-transformed. Higher scores of WRAT indicated a better performance

*CI* confidence interval, *IL* interleukin, *TNF* tumor necrosis factor, *WRAT* Wide Range Achievement Test

**Table 4:** Gestational cytokine concentration in the 2nd and 3rd trimesters and children’s neuropsychological functioning at 7 years (*n* = 1128)

	Bender Gestalt score		Goodenough-Harris Drawing score		Tactile Finger Recognition score	
	*B*	99% CI	*B*	99% CI	*B*	99% CI
IL-1β	0.003	−0.04, 0.04	−0.01	−0.41, 0.39	0.01	−0.01, 0.02
IL-8	−0.01	−0.05, 0.03	0.51	0.11, 0.90	0.02	0.01, 0.04
IL-6	0.03	−0.02, 0.08	0.04	−0.57, 0.64	0.01	−0.01, 0.02
IL-10	0.04	−0.01, 0.10	−0.04	−0.38, 0.60	0.01	−0.004, 0.03
TNF-α	0.12	0.001, 0.24	−1.28	−2.49, −0.07	0.002	−0.02, 0.03

Models are adjusted for child sex and age, and maternal race, education, age, socioeconomic disadvantage, smoking in pregnancy, history of treatment for psychiatric disorders, body mass index, and preeclampsia, and gestational diabetes

Effect sizes are reported per log unit increase in the cumulative cytokine concentration (pg/mL) of each cytokine during gestation

Bender Gestalt Scores and Tactile Finger Recognition Score were log-transformed

Higher scores in Bender Gestalt test indicated more problems. Higher scores in Goodenough-Harris Drawing test and Tactile Finger Recognition test indicated a better performance

*CI* confidence interval, *IL* interleukin, *TNF* tumor necrosis factor

Results of the categorical analyses of cytokine exposure revealed no association specific to either end of the cytokine distributions (data not shown). Finally, we found no evidence for sex differences in the associations between gestational inflammatory markers and 7-year neurocognitive function (data not shown).

## Discussion

We found that higher cumulative concentrations of TNF-α and lower concentrations of IL-8 throughout the 2nd and 3rd trimesters were associated with poorer age 7 neurocognitive functioning. Higher TNF-α concentration was associated with lower IQ, poorer cognitive performance, and higher problem scores related to visual-motor maturity, whereas children exposed prenatally to lower levels of IL-8 had *poorer* scores for cognitive performance and motor function. Cumulative concentrations of cytokines had large means and standard deviations (Table 1). The observed effect in outcomes (e.g., IQ) per unit increase in the exposure (concentrations of each cytokine or cytokine ratio in the log scale) thus likely represents relatively small effect sizes.

Maternal immune activation during pregnancy has been directly linked to offspring neurodevelopment in preclinical studies with longitudinal monitoring of behavioral and brain processes during the course of neurodevelopment. For example, structural brain abnormalities (e.g., in the dopaminergic system) and behavioral alterations (e.g., in learning and memory paradigms or social behavior) are observed in animal models of schizophrenia after gestational exposure to immune activation^31^, consistent with our prior work^11^. Regional brain alterations in the serotonergic system and autism-like behavioral changes are also shown in rodents after gestational administration of pro-inflammatory cytokines though information on cytokine content in the brain is limited^32^. While animal models provide valuable insight into the molecular mechanisms of gestational immune activation and brain abnormalities, translation of these findings to humans are limited due to differences in the severity and timing of gestational immune activation and species-specific differences in developmental trajectories and behavioral phenotypes in humans and in animals, among other factors^33,34^. Ecological studies of infectious exposures have provided indirect evidence of an association between elevated gestational inflammation and neuropsychiatric disorders among offspring^35^. One large nested case-control study of systemic inflammation and infection in the form of prenatal fever found an association with increased risk of autism, with risk increasing over 3-fold in offspring of mothers reporting three or more fever episodes in the 2nd and 3rd trimesters^5^. In some recent studies directly evaluating the biomarkers of gestational inflammation, increased rates of neuropsychiatric disorders have been observed^13,36,37^. However, until now there have been no direct tests of the involvement of gestational inflammation in neurocognitive development during childhood, which, given evidence of the neurocognitive precursors of psychiatric disorders^14^, plays a plausible role in mediating the association between gestational inflammation and offspring risk of neuropsychiatric disorders. We addressed this gap in knowledge by measuring inflammatory markers in stored prenatal sera from pregnant women and linking these markers to neurocognitive function.

Reports in animal models suggest that cytokines, in particular, T helper 1 cytokines such as TNF-α, can pass through the placental–fetal barrier to reach fetal brain^38^; other studies show, in addition, indirect effects of certain cytokines on the developing fetus through induction of placental dysfunction^39^. Whether acting directly or indirectly, many cytokines are expressed in the placenta and play a crucial role in regulation of the maternal–fetal immune interface to prevent maternal rejection of the fetus. At the same time, a number of these cytokines are known to affect differentiation and growth of neural cells in addition to their effect on immune cell populations^40^. Cytokines such as TNF-α have been shown to modulate the survival and growth of neuronal and glial cells^41^. TNF-α also induces IL-6 synthesis in astrocytes and enhances release of nerve growth factor^42^. In vitro examination of neural cells exposed to TNF-α has shown a decrease in the development of dendrites on embryonic cortical neurons, suggesting a putative mechanism for neurodevelopmental influences of TNF-α^43^. Based on this evidence, even minor deviation in normal TNF-α levels during pregnancy might be associated with adverse fetal brain development. Our findings suggest that intrauterine exposure to elevated levels of TNF-α may trigger some of the brain abnormalities that underlie appearance of neurocognitive impairment in childhood.

Contradictory reports exist concerning the influence of fetal sex on circulating gestational cytokine levels such as TNF-α. While one study showed no differences in serum TNF-α between women carrying male and females fetuses only reporting higher stimulated production of pro-inflammatory cytokines in women carrying female fetuses^44^, another reported that women with male fetuses had higher pro-inflammatory cytokine levels during mid to late pregnancy compared to women carrying female fetuses^45^. In the NEFS cohort, female offspring exposed to lower gestational levels of TNF-α were more likely than male offspring to develop schizophrenia^11^ and depression^13^. We found no significant sex differences in the association between gestational TNF-α and offspring neurocognitive function outcomes in childhood. One speculative explanation is that sex differences in neurocognitive outcomes may not manifest until post-puberty. Timing of the measurement of TNF-α might also play a role in detecting sex differences in child neurocognitive outcomes.

For IL-8, we found that lower concentrations were associated with *more impaired* cognitive performance and motor function. In a large nested case-control study, mothers of children with ASD had no differences in IL-8 levels during mid-gestation compared to controls^12^. Interestingly, when the study was restricted to children with ASD who did not have cognitive impairments, lower levels of IL-8 were associated with increased risk of ASD in children compared to general population controls. In contrast, in another prospective study, higher gestational IL-8 was associated with increased risk of schizophrenia in adults offspring^46^. Similarly, in 17 patients with schizophrenia, associations were shown between higher gestational IL-8 and multiple structural brain changes consistent with the symptom constellation of schizophrenia^47^. Given the differences reported in the directions of associations between these immune molecules and neuropsychiatric outcomes, the impact of cytokines might be brain-region and brain-function specific. Our outcome measures taken at age 7 may represent more subtle findings than those consistent with major neuropsychiatric disorders. Replication studies are needed to confirm this.

We did not observe any associations between IL-6, IL-10, and IL-1β and neurocognitive function. Some studies have shown a link between gestational levels of IL-6 and offspring ASD, psychosis, and developmental delays^11,12^. In contrast, Abdallah et al. showed no association between IL-6 measured in amniotic fluid and ASD^48^. In in vitro studies, IL-6 and IL-1β are shown to significantly reduce dendrite development and complexity of developing cortical neurons (pathologic findings consistent with features of schizophrenia)^43^, whereas some epidemiological studies reported no association of IL-6 and IL-1β and schizophrenia^46^. Findings regarding the relationship between gestational IL-10 and child neurodevelopment have also been conflicting^37,48^. It is noteworthy that most of these studies investigated the link between gestational cytokine levels and neuropsychiatric disorders. The effect of elevated cytokines on brain development might only be prominent in children with severe developmental delays or in those who later onset with major neuropsychiatric disorders.

This study had several strengths including a longitudinal design and a large sample size, repeated measurement of cytokines, broad control of potential confounders, and direct assessment of neurocognitive skills across broad domains. However, serum samples were stored for a long period of time and degradation of cytokines was possible. We do not expect degradation within samples to be differential according to maternal or fetal factors or to be associated with later child development. All samples were stored under similar conditions and underwent similar number of freeze–thaw cycles prior to running in cytokine assays. Additionally, we previously reported that the medians and ranges of cytokines (e.g., IL-8 and TNF-α) in the subsample of the CPP are comparable to those observed in more recent studies^16,49^. Among approximately 40% of participants, only one serum sample was available, potentially influencing our estimates of cumulative cytokine levels within the population. We included assays of five cytokines previously linked to psychopathologic disorders among offspring; however, recent preclinical studies suggest a role of additional cytokines not included in this analysis, including the pro-inflammatory molecule, IL-17^50^. We did not have sufficient numbers of samples from individual pregnancy within each trimester to examine the potential trimester-specific effects of exposures to inflammation.

In summary, we found associations between 2nd and 3rd trimester TNF-α and IL-8 and offspring neurocognitive functioning in children up to age 7. The opposing direction of association observed between TNF-α and IL-8 and childhood outcomes suggests that gestational inflammation and markers of immune function may have pleiotropic effects across domains of neurocognitive function in childhood. Impairments in neurocognitive functioning are closely related to childhood psychiatric disorders such as ASD, ADHD, and conduct disorder. These psychopathological disorders in children are multifactorial. Yet, our findings point to a potential role of immune processes in abnormal neurocognitive development of children. Future studies with repeated measurements of cytokines during pregnancy and across all trimesters of pregnancy will help to define the natural variation in cytokine levels throughout gestation and determine whether associations of maternal gestational immune function with offspring neurocognitive development are trimester specific.

## Electronic supplementary material

Supplementary Tables

## References

[1] Shi L (2009). Activation of the maternal immune system alters cerebellar development in the offspring. Brain Behav. Immun..

[2] Instanes JT (2015). Attention-Deficit/Hyperactivity Disorder in offspring of mothers with inflammatory and immune system diseases. Biol. Psychiatry.

[3] Estes ML, McAllister AK (2016). Maternal immune activation: implications for neuropsychiatric disorders. Science.

[4] Knuesel I (2014). Maternal immune activation and abnormal brain development across CNS disorders. Nat. Rev. Neurosci..

[5] Hornig M (2017). Prenatal fever and autism risk. Mol. Psychiatry.

[6] Brown AS (2014). Elevated maternal C-reactive protein and autism in a national birth cohort. Mol. Psychiatry.

[7] Zerbo O (2016). Maternal mid-pregnancy C-reactive protein and risk of autism spectrum disorders: the early markers for autism study. Transl. Psychiatry.

[8] Blackmore ER (2011). Psychiatric symptoms and proinflammatory cytokines in pregnancy. Psychosom. Med..

[9] Shimaoka Y (2000). Changes in cytokine production during and after normal pregnancy. Am. J. Reprod. Immunol..

[10] Kruse N (2000). Variations in cytokine mRNA expression during normal human pregnancy. Clin. Exp. Immunol..

[11] Goldstein JM (2014). Prenatal maternal immune disruption and sex-dependent risk for psychoses. Psychol. Med..

[12] Jones KL (2017). Autism with intellectual disability is associated with increased levels of maternal cytokines and chemokines during gestation. Mol. Psychiatry.

[13] Gilman SE (2016). Prenatal immune programming of the sex-dependent risk for major depression. Transl. Psychiatry.

[14] Hung GC (2016). Cognitive ability in childhood and the chronicity and suicidality of depression. Br. J. Psychiatry.

[15] Golan HM, Lev V, Hallak M, Sorokin Y, Huleihel M (2005). Specific neurodevelopmental damage in mice offspring following maternal inflammation during pregnancy. Neuropharmacology.

[16] Gilman SE (2017). Socioeconomic disadvantage, gestational immune activity, and neurodevelopment in early childhood. Proc. Natl Acad. Sci. USA.

[17] Mitchell RH, Goldstein BI (2014). Inflammation in children and adolescents with neuropsychiatric disorders: a systematic review. J. Am. Acad. Child Adolesc. Psychiatry.

[18] Szelenyi J, Vizi ES (2007). The catecholamine cytokine balance: interaction between the brain and the immune system. Ann. N. Y. Acad. Sci..

[19] Schobitz B, de Kloet ER, Sutanto W, Holsboer F (1993). Cellular localization of interleukin 6 mRNA and interleukin 6 receptor mRNA in rat brain. Eur. J. Neurosci..

[20] Vignali DA (2000). Multiplexed particle-based flow cytometric assays. J. Immunol. Methods.

[21] Wechsler D (1949). Manual for the Wechsler Intelligence Scale for Children.

[22] Jastak, J. F. & Jastak, S. R. *The Wide Range Achievement Test: Manual of Instructions* (Guidance Associates of Delaware, Delaware, 1965).

[23] Bender, L. *Instructions for the Use of Visual Motor Gestalt Test: Cards and Manual of Instructions* (American Orthopsychiatric Association, Itsaca, IL, 1946).

[24] Reitan, R. M. *Manual for Administration of Neuropsychological Test Batteries for Adults and Children* (Neuropsychology Laboratory, Indiana University medical Center, Indianapolis, IN, 1979).

[25] Scott LH (1981). Measuring intelligence with the Goodenough-Harris Drawing Test. Psychol. Bull..

[26] Chin-Lun Hung G (2015). Socioeconomic disadvantage and neural development from infancy through early childhood. Int. J. Epidemiol..

[27] Spiegelman D, McDermott A, Rosner B (1997). Regression calibration method for correcting measurement-error bias in nutritional epidemiology. Am. J. Clin. Nutr..

[28] Laird NM, Ware JH (1982). Random-effects models for longitudinal data. Biometrics.

[29] Austgulen R, Lien E, Liabakk NB, Jacobsen G, Arntzen KJ (1994). Increased levels of cytokines and cytokine activity modifiers in normal pregnancy. Eur. J. Obstet. Gynecol. Reprod. Biol..

[30] Koks N (2016). Maternal C-reactive protein concentration in early pregnancy and child autistic traits in the general population. Paediatr. Perinat. Epidemiol..

[31] Piontkewitz Y, Arad M, Weiner I (2012). Tracing the development of psychosis and its prevention: what can be learned from animal models. Neuropharmacology.

[32] Careaga M, Murai T, Bauman MD (2017). Maternal immune activation and autism spectrum disorder: from rodents to nonhuman and human primates. Biol. Psychiatry.

[33] Meyer U (2006). The time of prenatal immune challenge determines the specificity of inflammation-mediated brain and behavioral pathology. J. Neurosci..

[34] Han X, Li N, Meng Q, Shao F, Wang W (2011). Maternal immune activation impairs reversal learning and increases serum tumor necrosis factor-alpha in offspring. Neuropsychobiology.

[35] Mednick SA, Machon RA, Huttunen MO, Bonett D (1988). Adult schizophrenia following prenatal exposure to an influenza epidemic. Arch. Gen. Psychiatry.

[36] Buka SL (2001). Maternal cytokine levels during pregnancy and adult psychosis. Brain Behav. Immun..

[37] Goines PE (2011). Increased midgestational IFN-gamma, IL-4 and IL-5 in women bearing a child with autism: a case-control study. Mol. Autism.

[38] Bell MJ, Hallenbeck JM, Gallo V (2004). Determining the fetal inflammatory response in an experimental model of intrauterine inflammation in rats. Pediatr. Res..

[39] Ashdown H (2006). The role of cytokines in mediating effects of prenatal infection on the fetus: implications for schizophrenia. Mol. Psychiatry.

[40] Deverman BE, Patterson PH (2009). Cytokines and CNS development. Neuron.

[41] Cacci E, Ajmone-Cat MA, Anelli T, Biagioni S, Minghetti L (2008). In vitro neuronal and glial differentiation from embryonic or adult neural precursor cells are differently affected by chronic or acute activation of microglia. Glia.

[42] Galve-Roperh I (1998). Evidence for the lack of involvement of sphingomyelin hydrolysis in the tumor necrosis factor-induced secretion of nerve growth factor in primary astrocyte cultures. J. Neurochem..

[43] Gilmore JH, Fredrik Jarskog L, Vadlamudi S, Lauder JM (2004). Prenatal infection and risk for schizophrenia: IL-1beta, IL-6, and TNFalpha inhibit cortical neuron dendrite development. Neuropsychopharmacology.

[44] Mitchell AM, Palettas M, Christian LM (2017). Fetal sex is associated with maternal stimulated cytokine production, but not serum cytokine levels, in human pregnancy. Brain Behav. Immun..

[45] Enninga EA, Nevala WK, Creedon DJ, Markovic SN, Holtan SG (2015). Fetal sex-based differences in maternal hormones, angiogenic factors, and immune mediators during pregnancy and the postpartum period. Am. J. Reprod. Immunol..

[46] Brown AS (2004). Elevated maternal interleukin-8 levels and risk of schizophrenia in adult offspring. Am. J. Psychiatry.

[47] Ellman LM (2010). Structural brain alterations in schizophrenia following fetal exposure to the inflammatory cytokine interleukin-8. Schizophr. Res..

[48] Abdallah MW (2013). Amniotic fluid inflammatory cytokines: potential markers of immunologic dysfunction in autism spectrum disorders. World J. Biol. Psychiatry.

[49] Ferguson KK, Meeker JD, McElrath TF, Mukherjee B, Cantonwine DE (2017). Repeated measures of inflammation and oxidative stress biomarkers in preeclamptic and normotensive pregnancies. Am. J. Obstet. Gynecol..

[50] Choi GB (2016). The maternal interleukin-17a pathway in mice promotes autism-like phenotypes in offspring. Science.

