# Supplementary material for: Gestational cytokine concentrations and neurocognitive development at 7 years
Source: Transl Psychiatry. 2018 Mar 13;8:64. doi: 10.1038/s41398-018-0112-z (PMC5847536; doi:10.1038/s41398-018-0112-z)
Supplement: Supplementary file 1 — Supplementary Tables [file 41398_2018_112_MOESM1_ESM.docx]

**Supplementary Table 1** Cytokine measures available in pregnancy

| **Count per pregnancy** | **IL-1β** | **IL-8** | **IL-6** | **IL-10** | **TNF-α** |
| --- | --- | --- | --- | --- | --- |
| 1 | 549 (40.2%) | 547 (40.0%) | 546 (40.0%) | 546 (40.0%) | 546 (40.0%) |
| 2 | 654 (47.9%) | 657 (48.1%) | 657 (48.1%) | 657 (48.1%) | 657 (48.1%) |
| 3 | 145 (10.6%) | 145 (10.6%) | 145 (10.6%) | 145 (10.6%) | 145 (10.6%) |
| 4 | 18 (1.3%) | 17 (1.2%) | 18 (1.3%) | 18 (1.3%) | 18 (1.3%) |
| **Gestational age at draw** | | | | | |
| Weeks 14-20 | 232 | 232 | 232 | 232 | 232 |
| Weeks 21-26 | 506 | 506 | 506 | 506 | 506 |
| Weeks 27-33 | 1009 | 1008 | 1010 | 1010 | 1010 |
| Weeks 34 | 99 | 99 | 99 | 99 | 99 |
| Week 35+ | 153 | 154 | 154 | 154 | 154 |
| Drawn at delivery | 364 | 364 | 365 | 365 | 365 |

*Interleukin: IL; Tumor Necrosis Factor: TNF*

**Supplementary Table 2** Bivariate correlation between cumulative cytokine levels (in the log scale of pg/ml concentrations) during pregnancy and continuous covariates (n=1366)

|  | 1 | 2 | 3 | 4 | 5 | 6 | 7 | 8 |
| --- | --- | --- | --- | --- | --- | --- | --- | --- |
| 1. IL-6 | - |  |  |  |  |  |  |  |
| 2. IL-8 | 0.30** | - |  |  |  |  |  |  |
| 3. IL-10 | 0.48** | 0.19** | - |  |  |  |  |  |
| 4. IL-1β | 0.40** | 0.17** | 0.29** | - |  |  |  |  |
| 5. TNF-α | 0.30** | 0.33** | 0.23** | 0.14** | - |  |  |  |
| 6. Maternal age at enrollment | 0.06* | 0.04 | -0.02 | 0.01 | 0.05* | - | - |  |
| 7. Pre-pregnancy body mass index | 0.08** | -0.04 | -0.05 | -0.03 | 0.01 | 0.21** |  |  |
| 8. Maternal years of education | 0.05 | 0.12** | 0.08** | 0.04 | 0.004 | 0.09** | -0.02 |  |
| 9. Maximum number of cigarette per day in pregnancy | -0.02 | 0.03 | 0.004 | -0.02 | 0.06* | -0.003 | -0.04 | -0.16** |

* Significant at p<0.05 (two-tailed), ** Significant at p<0.01 (two-tailed)

*Interleukin: IL; Tumor Necrosis Factor: TNF*

**Supplementary Table 3** Univariate associations of maternal cumulative cytokine levels with categorical covariates

|  | | IL-1β | IL-8 | IL-6 | IL-10 | TNF-α |
| --- | --- | --- | --- | --- | --- | --- |
| Race | |  |  |  |  |  |
|  | White | 3.37 (1.15) | 6.00 (1.17) | 3.31 (0.82) | 3.75 (0.71) | 4.46 (0.36) |
|  | Non-White | 3.26 (1.22) | 5.66 (1.11) | 3.35 (0.93) | 3.71 (0.77) | 4.40 (0.41) |
| Prenatal socioeconomic disadvantage | |  |  |  |  |  |
|  | 0-1 | 3.44 (1.15)* | 6.18 (1.15)** | 3.33 (0.79) | 3.80 (0.76)** | 4.46 (0.35) |
|  | 1.5-2.5 | 3.32 (1.16) | 5.87 (1.18) | 3.33 (0.87) | 3.72 (0.70) | 4.46 (0.39) |
|  | >=3 | 3.24 (1.18) | 5.49 (1.04) | 3.24 (0.87) | 3.65 (0.61) | 4.42 (0.37) |
| History of treatment for psychiatric disorders | |  |  |  |  |  |
|  | No | 3.36 (0.16) | 5.94 (1.16) | 3.31 (0.82) | 3.75 (0.73) | 4.45 (0.36) |
|  | Yes | 3.42 (0.14) | 6.11 (1.23) | 3.37 (0.91) | 3.72 (0.63) | 4.49 (0.37) |
| Preeclampsia | |  |  |  |  |  |
|  | No | 3.37 (0.16) | 5.96 (1.67) | 3.31 (0.83) | 3.75 (0.72) | 4.45 (0.36) |
|  | Yes | 2.93 (0.90) | 5.63 (1.27) | 3.37 (0.72) | 3.69 (0.64) | 4.47 (0.45) |
| Gestational diabetes | |  |  |  |  |  |
|  | No | 3.36 (0.16) | 5.96 (1.17)** | 3.31 (0.83) | 3.74 (0.72)* | 4.45 (0.37) |
|  | Yes | 3.50 (0.68) | 5.46 (0.37) | 3.29 (0.74) | 4.14 (0.56) | 4.50 (0.26) |
| Child sex | |  |  |  |  |  |
|  | Boy | 3.38 (1.20) | 5.98 (1.10) | 3.32 (0.80) | 3.77 (0.75) | 4.48 (0.32)** |
|  | Girl | 3.35 (1.12) | 5.95 (1.22) | 3.31 (0.85) | 3.73 (0.69) | 4.43 (0.39) |

Numbers are mean (SD) of cumulative cytokine levels in pregnancy (in log scale of pg/ml concentrations).

Independent sample *t*-test or analysis of variance was used to examine the associations of cumulative cytokine levels (in the log scale of pg/ml concentrations) with categorical covariates.

* Significant at p<0.05 (two-tailed), ** Significant at p<0.01 (two-tailed)

*Interleukin: IL; Tumor Necrosis Factor: TNF*

**Supplementary Table 4** Gestational cytokine levels in the 2^nd^ and 3^rd^ trimesters

|  | **N for Assays** |  | **Effect Estimate** | **Standard Error** |
| --- | --- | --- | --- | --- |
| **IL-1β** | 2363 | Intercept | 0.05 | 0.10 |
|  |  | GA, if <27 weeks | 0.02 | 0.01 |
|  |  | GA, if >27 weeks | 0.0003 | 0.02 |
|  |  | If provided sample around birth (yes/no) | -0.18 | 0.17 |
| **IL-8** | 2363 | Intercept | 2.70* | 0.09 |
|  |  | GA, if <27 weeks | -0.01 | 0.02 |
|  |  | GA, if >27 weeks | -0.03* | 0.01 |
|  |  | If provided sample around birth (yes/no) | 0.02 | 0.14 |
| **IL-6** | 2366 | Intercept | -0.07 | 0.08 |
|  |  | GA, if <27 weeks | -0.01 | 0.01 |
|  |  | GA, if >27 weeks | 0.0.03* | 0.01 |
|  |  | If provided sample around birth (yes/no) | 2.46* | 0.13 |
| **IL-10** | 2366 | Intercept | 0.61* | 0.06 |
|  |  | GA, if <27 weeks | 0.04* | 0.01 |
|  |  | GA, if >27 weeks | -0.01 | 0.01 |
|  |  | If provided sample around birth (yes/no) | 1.64* | 0.11 |
| **TNF-α** | 2366 | Intercept | 1.12* | 0.04 |
|  |  | GA, if <27 weeks | -0.02* | 0.01 |
|  |  | GA, if >27 weeks | 0.004 | 0.01 |
|  |  | If provided sample around birth (yes/no) | 0.18* | 0.08 |

* p <0.05, n for number of pregnant women included= 1366

*Gestational age at maternal serum sample draw: GA; Interleukin: IL; Tumor Necrosis Factor: TNF*

Derived from mixed effect models (with unstructured correlation matrix). Effect estimates define the slopes of cytokine concentrations (in log linear scale of pg/ml concentrations) in the second and third trimesters.

Maximum number of observation per subject = 4

Models were adjusted for an indicator variable (*D*) if samples were provided around birth, and effect estimates were calculated using the following

$$Y_{ij}= \boldsymbol{\beta}_{0}+\boldsymbol{\beta}_{1} GA \left( if<27 wks \right) + \boldsymbol{\beta}_{2} GA \left( if>27 wks \right) + \boldsymbol{\beta}_{3}D + b_{i}+\varepsilon_{ij}$$
